# *catena*-Poly[[triaquacopper(II)]-μ_2_-furan-2,5-dicarboxyl­ato-κ^4^ *O* ^1^,*O* ^2^:*O* ^2^,*O* ^2′^]

**DOI:** 10.1107/S1600536812010161

**Published:** 2012-03-17

**Authors:** Ya-Feng Li, Yue Gao, Yue Xu, Xiao-lin Qin, Wen-Yuan Gao

**Affiliations:** a School of Chemical Engineering, Changchun University of Technology, Changchun 130012, People’s Republic of China

## Abstract

In the title compound, [Cu(C_6_H_2_O_5_)(H_2_O)_3_]_*n*_, an infinite chain is formed along [001] by linking of the Cu(OH_2_)_3_O_4_ cluster with one carboxyl­ate group of the furan-2,5-dicarboxyl­ate ligand. Adjacent chains are linked by O_water_—H⋯O hydrogen-bonding inter­actions. The Cu(OH_2_)_3_O_4_ cluster displays a penta­gonal bipyrimadal geometry with two weak coordinations [Cu—O_furan_ = 2.790 (2) Å) and Cu—O_carboxyl­ate_ = 2.684 (2) Å] and two water mol­ecules located in axial positions.

## Related literature

For background to metalorganic framework materials, see: Chui *et al.* (1999 ▶); Corma *et al.* (2010 ▶); Ferey (2008 ▶); Li *et al.* (1999 ▶); Murray *et al.* (2009 ▶); Tranchemontagne *et al.* (2009 ▶). 
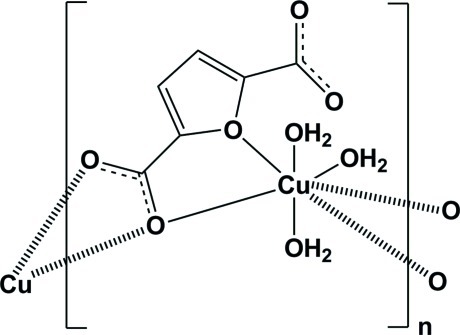


## Experimental

### 

#### Crystal data

[Cu(C_6_H_2_O_5_)(H_2_O)_3_]
*M* *_r_* = 271.66
Monoclinic, 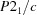

*a* = 7.0559 (14) Å
*b* = 15.040 (3) Å
*c* = 8.1578 (16) Å
β = 93.92 (3)°
*V* = 863.7 (3) Å^3^
*Z* = 4
Mo *K*α radiation
μ = 2.55 mm^−1^
*T* = 296 K
0.15 × 0.14 × 0.12 mm

#### Data collection

Rigaku R-AXIS RAPID diffractometer
Absorption correction: multi-scan (*ABSCOR*; Higashi, 1995 ▶) *T*_min_ = 0.701, *T*_max_ = 0.749
8400 measured reflections
1985 independent reflections
1656 reflections with *I* > 2σ(*I*)
*R*_int_ = 0.039

#### Refinement

*R*[*F* ^2^ > 2σ(*F* ^2^)] = 0.031
*wR*(*F* ^2^) = 0.070
*S* = 1.09
1985 reflections
154 parameters
10 restraints
H atoms treated by a mixture of independent and constrained refinement
Δρ_max_ = 0.43 e Å^−3^
Δρ_min_ = −0.44 e Å^−3^

### 

Data collection: *PROCESS-AUTO* (Rigaku, 1998 ▶); cell refinement: *PROCESS-AUTO*; data reduction: *CrystalStructure* (Rigaku/MSC, 2002) ▶; program(s) used to solve structure: *SHELXS97* (Sheldrick, 2008 ▶); program(s) used to refine structure: *SHELXL97* (Sheldrick, 2008 ▶); molecular graphics: *DIAMOND* (Brandenburg, 2000 ▶); software used to prepare material for publication: *SHELXL97*.

## Supplementary Material

Crystal structure: contains datablock(s) I, global. DOI: 10.1107/S1600536812010161/zj2064sup1.cif

Structure factors: contains datablock(s) I. DOI: 10.1107/S1600536812010161/zj2064Isup2.hkl

Additional supplementary materials:  crystallographic information; 3D view; checkCIF report

## Figures and Tables

**Table 1:** Hydrogen-bond geometry (Å, °)

*D*—H⋯*A*	*D*—H	H⋯*A*	*D*⋯*A*	*D*—H⋯*A*
O1*W*—H1*A*⋯O5^i^	0.81 (2)	2.04 (2)	2.843 (3)	173 (3)
O1*W*—H1*B*⋯O4^ii^	0.79 (2)	1.95 (2)	2.729 (3)	170 (3)
O2*W*—H2*A*⋯O5^iii^	0.82 (2)	1.91 (2)	2.690 (3)	161 (3)
O2*W*—H2*B*⋯O4^iv^	0.80 (2)	2.55 (3)	3.118 (3)	129 (3)
O3*W*—H3*A*⋯O4	0.81 (2)	1.90 (2)	2.704 (3)	171 (3)
O3*W*—H3*B*⋯O1^v^	0.80 (2)	1.92 (2)	2.715 (2)	172 (3)

Symmetry codes: (i) 
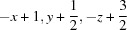
; (ii) 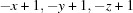; (iii) 
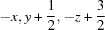
; (iv) 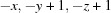; (v) 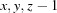.

**Fig. 1.**
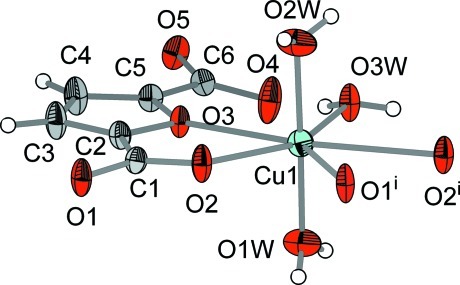
The unit cell of (I), showing the atomic labelling scheme and displacement ellipsoids at the 50% probability level. [Symmetry codes: (i) x, 1.5 - y, -0.5 + z.]

**Fig. 2.**
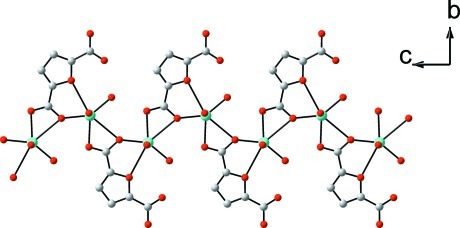
The stick plot of (I), displaying the infinite chain along (001) direction formed by linking the Cu with carboxyl of furan-2,5-dicarboxylate.

**Fig. 3.**
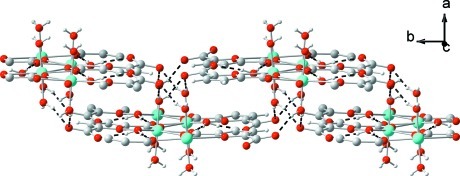
The ball-stick packing diagram of (I). The H-bond of OW—H···O holds together adjacent chains.

## supplementary crystallographic information

### Comment

During past decades, the MOF materials have being attracted huge attentions due to the applications including gas absorption and catalyst reactions(Murray, *et al.*, 2009; Corma, *et al.*, 2010). The more efforts have been focused on the MOF based on the phenyl ring with carboxyl groups (Chui, *et al.*, 1999; Li, *et al.*, 1999; Ferey, 2008; Tranchemontagne, *et al.*, 2009). Compared with phenyl ring with carboxyl groups, the 5-membered rings with carboxyl groups as the ligand are rarely studied. Recently, we utilize furan-2,5-dicarboxyl acid as the ligand to constructed the MOFs. In this work, chainlike compound, [Cu^.^(C_6_O_5_H_2_)^.^3H_2_O]_n_ (I), is synthesized.

The asymmetric unit of (I) comprises of one Cu(II) cation, one furan-2,5-dicarboxylate anion and three H_2_O (Fig.1). Cu cation is coordinated by three carboxyl O atoms, one oxygen of furan ring, and three water molecules of which two locate at the poles, exhibiting pentagonal bipyrimad geometry. It is necessarily noted that oxygen of furan ring (d_O—Cu_=2.790 (2) Å) and η^2^-oxygen of carboxyl (d_O—Cu_=2.684 (2) Å) are very weakly ligated to Cu cation. If excluding this two O atoms, Cu displays triganol bipyramid geometry and the chain property may not be changed. Only one carboxyl of furan-2,5-dicarboxylate involves in the formation of Cu polyhedron. The carboxyl shows µ_2_:η^1^,η^2^ mode.

Cu cations are linked by one carboxyl of furan-2,5-dicarboxylate to give rise to the infinite chain (Fig.2). The H-bond of OW—H···O holds together adjacent chains (Fig.3).

### Experimental

(I) was synthesized under solvothermal condition. In a typically route, furan-2,5-dicarboxyl acid (0.312 g, 2.0 mmol) and Cu(NO_3_)_2_^.^3H_2_O (0.48 g, 2.0 mmol) were dissolved in the mixture of EtOH (2.9 ml, 50 mmol) and DMF (3.9 ml, 50 mmol) under stirring. Then, the clear solution with molar ratio of 1 (furan-2,5-dicarboxyl acid): 1 (Cu(NO_3_)_2_^.^3H_2_O): 25 (EtOH): 25 (DMF) was tranferred into 23 ml autoclave and heated at 393 K for 24hrs. After naturally cooling to room temperature, blue block product was collected by filtration as a single phase.

### Refinement

Water H atoms were located in a difference Fourier map and were refined with O—H = 0.82 (2) Å, H···H = 1.37 (2) Å and *U*_iso_(H) = 1.2Ueq(O). The carbon H-atoms were placed in calculated positions (C—H = 0.93 Å) and were included in the refinement in the riding-model approximation, with *U*_iso_(H) = 1.2Ueq(C).

### Crystal data

**Table:**

[Cu(C_6_H_2_O_5_)(H_2_O)_3_]	*F*(000) = 548
*M**_r_* = 271.66	*D*_x_ = 2.089 Mg m^−^^3^
Monoclinic, *P*2_1_/*c*	Mo *K*α radiation, λ = 0.71073 Å
Hall symbol: -P 2ybc	Cell parameters from 2000 reflections
*a* = 7.0559 (14) Å	θ = 3.2–27.5°
*b* = 15.040 (3) Å	µ = 2.55 mm^−^^1^
*c* = 8.1578 (16) Å	*T* = 296 K
β = 93.92 (3)°	Block, light blue
*V* = 863.7 (3) Å^3^	0.15 × 0.14 × 0.12 mm
*Z* = 4	

### Data collection

**Table:**

Rigaku R-AXIS RAPID diffractometer	1985 independent reflections
Radiation source: fine-focus sealed tube	1656 reflections with *I* > 2σ(*I*)
Graphite monochromator	*R*_int_ = 0.039
Detector resolution: 10.00 pixels mm^-1^	θ_max_ = 27.5°, θ_min_ = 3.2°
ω scans	*h* = −9→9
Absorption correction: multi-scan (*ABSCOR*; Higashi, 1995)	*k* = −18→19
*T*_min_ = 0.701, *T*_max_ = 0.749	*l* = −10→10
8400 measured reflections	

### Refinement

**Table:**

Refinement on *F*^2^	Primary atom site location: structure-invariant direct methods
Least-squares matrix: full	Secondary atom site location: difference Fourier map
*R*[*F*^2^ > 2σ(*F*^2^)] = 0.031	Hydrogen site location: inferred from neighbouring sites
*wR*(*F*^2^) = 0.070	H atoms treated by a mixture of independent and constrained refinement
*S* = 1.09	*w* = 1/[σ^2^(*F*_o_^2^) + (0.0277*P*)^2^ + 0.660*P*] where *P* = (*F*_o_^2^ + 2*F*_c_^2^)/3
1985 reflections	(Δ/σ)_max_ = 0.001
154 parameters	Δρ_max_ = 0.43 e Å^−^^3^
10 restraints	Δρ_min_ = −0.44 e Å^−^^3^

### Special details

**Table:**

Geometry. All e.s.d.'s (except the e.s.d. in the dihedral angle between two l.s. planes) are estimated using the full covariance matrix. The cell e.s.d.'s are taken into account individually in the estimation of e.s.d.'s in distances, angles and torsion angles; correlations between e.s.d.'s in cell parameters are only used when they are defined by crystal symmetry. An approximate (isotropic) treatment of cell e.s.d.'s is used for estimating e.s.d.'s involving l.s. planes.
Refinement. Refinement of *F*^2^ against ALL reflections. The weighted *R*-factor *wR* and goodness of fit *S* are based on *F*^2^, conventional *R*-factors *R* are based on *F*, with *F* set to zero for negative *F*^2^. The threshold expression of *F*^2^ > σ(*F*^2^) is used only for calculating *R*-factors(gt) *etc*. and is not relevant to the choice of reflections for refinement. *R*-factors based on *F*^2^ are statistically about twice as large as those based on *F*, and *R*- factors based on ALL data will be even larger.

### Fractional atomic coordinates and isotropic or equivalent isotropic displacement parameters (Å^2^)

**Table:**

	*x*	*y*	*z*	*U*_iso_*/*U*_eq_	
Cu1	0.25359 (5)	0.68635 (2)	0.66688 (4)	0.02149 (11)	
O1	0.2757 (3)	0.66588 (12)	1.1767 (2)	0.0271 (5)	
O2	0.2608 (3)	0.69971 (11)	0.9141 (2)	0.0260 (4)	
O3	0.2629 (3)	0.52292 (11)	0.82901 (19)	0.0198 (4)	
O4	0.3038 (3)	0.42902 (12)	0.5442 (2)	0.0370 (5)	
O5	0.2282 (3)	0.30371 (11)	0.6657 (2)	0.0263 (4)	
C1	0.2663 (4)	0.64357 (16)	1.0277 (3)	0.0183 (5)	
C2	0.2593 (4)	0.54734 (15)	0.9908 (3)	0.0188 (5)	
C3	0.2454 (5)	0.47559 (17)	1.0867 (3)	0.0288 (6)	
H3	0.2409	0.4750	1.2003	0.035*	
C4	0.2389 (5)	0.40085 (17)	0.9808 (3)	0.0285 (6)	
H4	0.2283	0.3416	1.0116	0.034*	
C5	0.2511 (4)	0.43186 (15)	0.8272 (3)	0.0192 (5)	
C6	0.2608 (4)	0.38628 (16)	0.6674 (3)	0.0198 (5)	
O1W	0.5258 (3)	0.67555 (14)	0.6763 (2)	0.0320 (5)	
H1A	0.588 (4)	0.7150 (17)	0.721 (4)	0.038*	
H1B	0.573 (4)	0.6500 (19)	0.605 (3)	0.038*	
O2W	−0.0197 (3)	0.69352 (15)	0.6594 (3)	0.0356 (5)	
H2A	−0.060 (5)	0.7333 (18)	0.716 (3)	0.043*	
H2B	−0.074 (5)	0.690 (2)	0.570 (2)	0.043*	
O3W	0.2223 (3)	0.60185 (12)	0.4812 (2)	0.0261 (4)	
H3A	0.258 (4)	0.5519 (10)	0.503 (3)	0.031*	
H3B	0.245 (4)	0.6171 (18)	0.391 (2)	0.031*	

### Atomic displacement parameters (Å^2^)

**Table:**

	*U*^11^	*U*^22^	*U*^33^	*U*^12^	*U*^13^	*U*^23^
Cu1	0.02457 (18)	0.02215 (17)	0.01807 (16)	0.00066 (14)	0.00374 (11)	−0.00597 (13)
O1	0.0528 (14)	0.0170 (9)	0.0118 (8)	−0.0027 (8)	0.0048 (8)	−0.0024 (7)
O2	0.0502 (13)	0.0154 (8)	0.0128 (8)	0.0014 (8)	0.0053 (8)	0.0000 (7)
O3	0.0368 (11)	0.0108 (8)	0.0120 (7)	−0.0012 (7)	0.0037 (7)	−0.0012 (7)
O4	0.0747 (17)	0.0181 (9)	0.0202 (9)	0.0038 (10)	0.0169 (10)	0.0004 (8)
O5	0.0432 (13)	0.0127 (9)	0.0237 (9)	−0.0066 (8)	0.0073 (8)	−0.0050 (7)
C1	0.0236 (14)	0.0162 (11)	0.0156 (11)	0.0012 (10)	0.0041 (9)	−0.0006 (10)
C2	0.0276 (14)	0.0152 (11)	0.0138 (10)	0.0013 (10)	0.0019 (9)	−0.0023 (10)
C3	0.053 (2)	0.0201 (12)	0.0129 (12)	−0.0026 (13)	0.0030 (11)	−0.0011 (11)
C4	0.0534 (19)	0.0132 (11)	0.0187 (12)	−0.0006 (12)	0.0014 (12)	0.0001 (11)
C5	0.0272 (13)	0.0109 (10)	0.0196 (11)	−0.0008 (10)	0.0018 (9)	−0.0026 (10)
C6	0.0236 (13)	0.0173 (12)	0.0184 (11)	0.0023 (10)	0.0014 (9)	−0.0021 (10)
O1W	0.0244 (11)	0.0360 (12)	0.0357 (11)	0.0011 (9)	0.0028 (9)	−0.0173 (9)
O2W	0.0238 (11)	0.0479 (13)	0.0353 (11)	0.0004 (10)	0.0025 (8)	−0.0235 (11)
O3W	0.0484 (13)	0.0161 (8)	0.0144 (8)	0.0038 (9)	0.0057 (8)	0.0012 (7)

### Geometric parameters (Å, º)

**Table:**

Cu1—O1W	1.924 (2)	C2—C3	1.340 (4)
Cu1—O2W	1.928 (2)	C3—C4	1.416 (4)
Cu1—O3W	1.9782 (18)	C3—H3	0.9300
Cu1—O2	2.0243 (17)	C4—C5	1.345 (3)
Cu1—O1^i^	2.2289 (18)	C4—H4	0.9300
O1—C1	1.258 (3)	C5—C6	1.479 (3)
O1—Cu1^ii^	2.2289 (18)	C6—O4	1.248 (3)
O2—C1	1.252 (3)	O1W—H1A	0.809 (17)
O3—C2	1.372 (3)	O1W—H1B	0.791 (17)
O3—C5	1.372 (3)	O2W—H2A	0.816 (17)
O4—C6	1.248 (3)	O2W—H2B	0.802 (17)
O5—C6	1.263 (3)	O3W—H3A	0.807 (15)
C1—C2	1.478 (3)	O3W—H3B	0.799 (16)
			
O1W—Cu1—O2W	178.29 (10)	C4—C3—H3	126.8
O1W—Cu1—O3W	92.01 (9)	C5—C4—C3	106.9 (2)
O2W—Cu1—O3W	87.28 (9)	C5—C4—H4	126.5
O1W—Cu1—O2	90.67 (9)	C3—C4—H4	126.5
O2W—Cu1—O2	89.03 (9)	C4—C5—O3	110.1 (2)
O3W—Cu1—O2	145.16 (7)	C4—C5—C6	132.1 (2)
O1W—Cu1—O1^i^	90.89 (8)	O3—C5—C6	117.8 (2)
O2W—Cu1—O1^i^	90.73 (9)	O4—C6—O5	123.5 (2)
O3W—Cu1—O1^i^	132.21 (7)	O4—C6—O5	123.5 (2)
O2—Cu1—O1^i^	82.44 (6)	O4—C6—C5	120.0 (2)
C1—O1—Cu1^ii^	103.45 (15)	O4—C6—C5	120.0 (2)
C1—O2—Cu1	131.89 (15)	O5—C6—C5	116.5 (2)
C2—O3—C5	105.81 (17)	Cu1—O1W—H1A	118 (2)
O2—C1—O1	122.1 (2)	Cu1—O1W—H1B	119 (2)
O2—C1—C2	120.7 (2)	H1A—O1W—H1B	116 (3)
O1—C1—C2	117.2 (2)	Cu1—O2W—H2A	114 (2)
C3—C2—O3	110.7 (2)	Cu1—O2W—H2B	116 (3)
C3—C2—C1	132.2 (2)	H2A—O2W—H2B	113 (3)
O3—C2—C1	117.1 (2)	Cu1—O3W—H3A	114.5 (15)
C2—C3—C4	106.5 (2)	Cu1—O3W—H3B	120 (2)
C2—C3—H3	126.8	H3A—O3W—H3B	113 (2)
			
O1W—Cu1—O2—C1	−84.2 (2)	C1—C2—C3—C4	178.1 (3)
O2W—Cu1—O2—C1	94.1 (2)	C2—C3—C4—C5	0.6 (4)
O3W—Cu1—O2—C1	10.3 (3)	C3—C4—C5—O3	−0.7 (3)
O1^i^—Cu1—O2—C1	−175.0 (3)	C3—C4—C5—C6	176.7 (3)
Cu1—O2—C1—O1	177.90 (19)	C2—O3—C5—C4	0.5 (3)
Cu1—O2—C1—C2	−3.0 (4)	C2—O3—C5—C6	−177.2 (2)
Cu1^ii^—O1—C1—O2	4.6 (3)	O4—O4—C6—O5	0.0 (3)
Cu1^ii^—O1—C1—C2	−174.55 (19)	O4—O4—C6—C5	0.0 (3)
C5—O3—C2—C3	−0.2 (3)	C4—C5—C6—O4	−167.8 (3)
C5—O3—C2—C1	−178.8 (2)	O3—C5—C6—O4	9.3 (4)
O2—C1—C2—C3	−172.9 (3)	C4—C5—C6—O4	−167.8 (3)
O1—C1—C2—C3	6.3 (5)	O3—C5—C6—O4	9.3 (4)
O2—C1—C2—O3	5.4 (4)	C4—C5—C6—O5	10.6 (5)
O1—C1—C2—O3	−175.4 (2)	O3—C5—C6—O5	−172.3 (2)
O3—C2—C3—C4	−0.2 (3)		

Symmetry codes: (i) *x*, −*y*+3/2, *z*−1/2; (ii) *x*, −*y*+3/2, *z*+1/2.

### Hydrogen-bond geometry (Å, º)

**Table:**

*D*—H···*A*	*D*—H	H···*A*	*D*···*A*	*D*—H···*A*
O1*W*—H1*A*···O5^iii^	0.81 (2)	2.04 (2)	2.843 (3)	173 (3)
O1*W*—H1*B*···O4^iv^	0.79 (2)	1.95 (2)	2.729 (3)	170 (3)
O2*W*—H2*A*···O5^v^	0.82 (2)	1.91 (2)	2.690 (3)	161 (3)
O2*W*—H2*B*···O4^vi^	0.80 (2)	2.55 (3)	3.118 (3)	129 (3)
O3*W*—H3*A*···O4	0.81 (2)	1.90 (2)	2.704 (3)	171 (3)
O3*W*—H3*B*···O1^vii^	0.80 (2)	1.92 (2)	2.715 (2)	172 (3)

Symmetry codes: (iii) −*x*+1, *y*+1/2, −*z*+3/2; (iv) −*x*+1, −*y*+1, −*z*+1; (v) −*x*, *y*+1/2, −*z*+3/2; (vi) −*x*, −*y*+1, −*z*+1; (vii) *x*, *y*, *z*−1.
